# Supplementary material for: In Vitro Modeling as a Tool for Testing Therapeutics for Spinal Muscular Atrophy and IGHMBP2-Related Disorders
Source: Biology (Basel). 2023 Jun 16;12(6):867. doi: 10.3390/biology12060867 (PMC10295315; doi:10.3390/biology12060867)
Supplement: Supplementary file 1 [file biology-12-00867-s001.zip › biology-2349688-Figure S1/western blot.pdf]

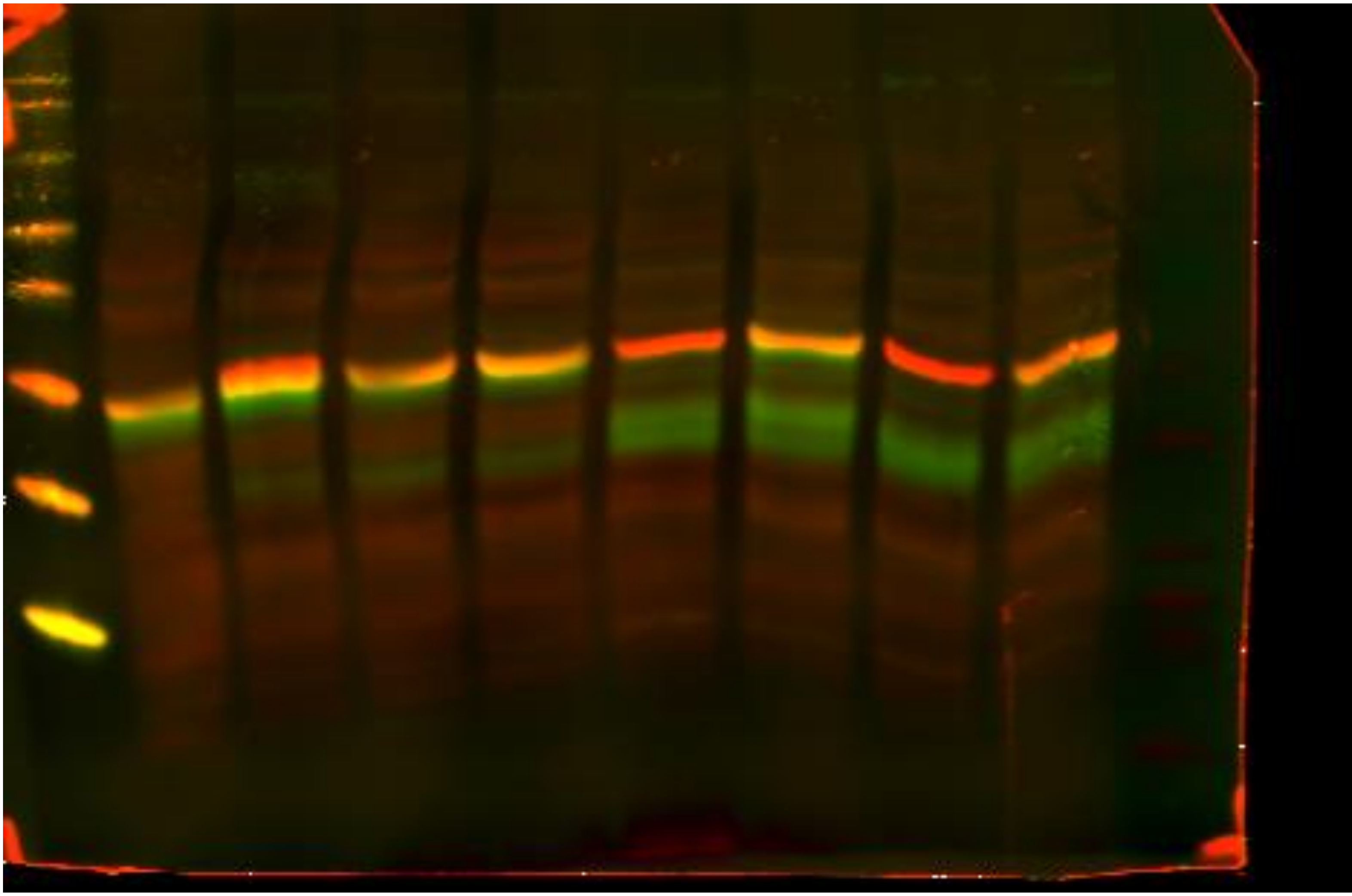

Lane 1: Control 1
Lane 2: Control 1 +AAV9.SMN
Lane 3: Control 2
Lane 4: Control 2+ AAV9.SMN
Lane 5: SMA patient 1
Lane 6: SMA patient 1+ AAV9.SMN
Lane7: SMA Patient 2
Lane 8: SMA patient 2+AAV9.SMN

SMN: Green (SMN band at 38kDA)
GAPDH: Red (Band at 37kDA)
