# Supplementary figures and images for: In Vitro Modeling as a Tool for Testing Therapeutics for Spinal Muscular Atrophy and IGHMBP2-Related Disorders
Source: Biology (Basel). 2023 Jun 16;12(6):867. doi: 10.3390/biology12060867 (PMC10295315; doi:10.3390/biology12060867)

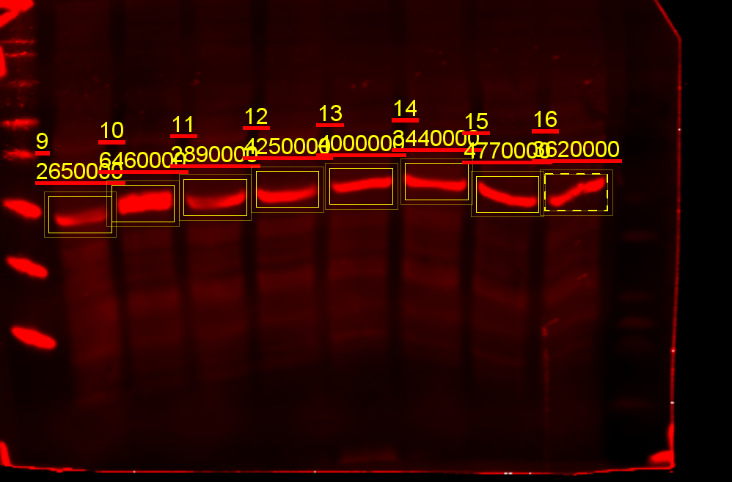

Supplement: Supplementary file 1 [file biology-12-00867-s001.zip › biology-2349688-Figure S1/GAPDH (RED).tif]

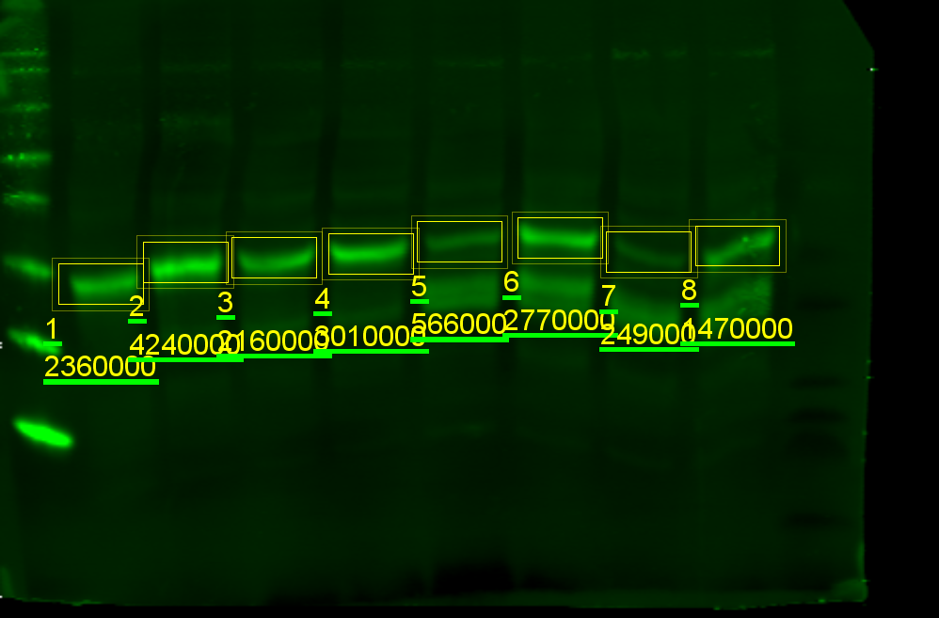

Supplement: Supplementary file 1 [file biology-12-00867-s001.zip › biology-2349688-Figure S1/SMN (GREEN).tif]

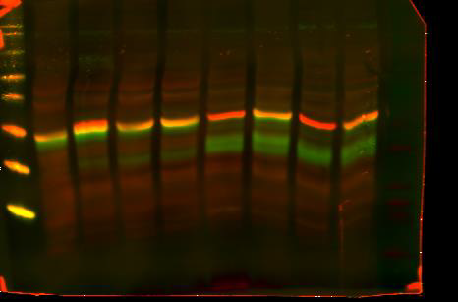

Supplement: Supplementary file 1 [file biology-12-00867-s001.zip › biology-2349688-Figure S1/western blot complete.tiff]
